# A SARS-CoV-2 neutralizing antibody discovery by single cell sequencing and molecular modeling

**DOI:** 10.7555/JBR.36.20220221

**Published:** 2022-12-12

**Authors:** Zheyue Wang, Qi Tang, Bende Liu, Wenqing Zhang, Yufeng Chen, Ningfei Ji, Yan Peng, Xiaohui Yang, Daixun Cui, Weiyu Kong, Xiaojun Tang, Tingting Yang, Mingshun Zhang, Xinxia Chang, Jin Zhu, Mao Huang, Zhenqing Feng

**Affiliations:** 1 National Health Commission Key Laboratory of Antibody Technique, Jiangsu Province Engineering Research Center of Antibody Drug, Department of Pathology, Nanjing Medical University, Nanjing, Jiangsu 211166, China; 2 Department of Cardiology, the First People's Hospital of Jiangxia District, Wuhan, Hubei 430299, China; 3 Department of Respiratory and Critical Care Medicine, the First Affiliated Hospital of Nanjing Medical University, Nanjing, Jiangsu 210029, China; 4 Department of Rheumatology and Immunology, the Affiliated Drum Tower Hospital of Nanjing University Medical School, Nanjing, Jiangsu 210008, China; 5 Huadong Medical Institute of Biotechniques, Nanjing, Jiangsu 210028, China; 6 Jiangsu Key Lab of Cancer Biomarkers, Prevention and Treatment, Collaborative Innovation Center for Cancer Personalized Medicine, Nanjing Medical University, Nanjing, Jiangsu 211166, China

**Keywords:** SARS-CoV-2, neutralizing antibody, single B cell BCR sequencing, molecular modeling

## Abstract

Although vaccines have been developed, mutations of SARS-CoV-2, especially the dominant B.1.617.2 (delta) and B.1.529 (omicron) strains with more than 30 mutations on their spike protein, have caused a significant decline in prophylaxis, calling for the need for drug improvement. Antibodies are drugs preferentially used in infectious diseases and are easy to get from immunized organisms. The current study combined molecular modeling and single memory B cell sequencing to assess candidate sequences before experiments, providing a strategy for the fabrication of SARS-CoV-2 neutralizing antibodies. A total of 128 sequences were obtained after sequencing 196 memory B cells, and 42 sequences were left after merging extremely similar ones and discarding incomplete ones, followed by homology modeling of the antibody variable region. Thirteen candidate sequences were expressed, of which three were tested positive for receptor binding domain recognition but only one was confirmed as having broad neutralization against several SARS-CoV-2 variants. The current study successfully obtained a SARS-CoV-2 antibody with broad neutralizing abilities and provided a strategy for antibody development in emerging infectious diseases using single memory B cell BCR sequencing and computer assistance in antibody fabrication.

## Introduction

The SARS-CoV-2 pandemic broke out in late 2019 and resulted in six million deaths and 628 million infections by the end of October 2022, according to the World Health Organization (WHO). Sharing 82% of its sequence with the SARS virus^[1]^, SARS-CoV-2 is an RNA virus that invades host cells by binding to angiotensin converting enzyme 2 (ACE2) with its receptor binding domain (RBD) within its spike (S) protein^[2]^. New variants of SARS-CoV-2 have emerged one by one: from the first variant B.1.1.7 to the latest B.1.1.529 (omicron), which had 15 mutations within RBD and were able to escape from neutralizing antibodies^[3]^. The three-year pandemic saw a dominant shift from SARS-CoV-2 wild type (WT) strain to the variant B.1.617.2 (delta) that was again followed by the omicron, resulting in the need for faster production and broader reactivity drugs, as current vaccines are unable to protect people from infection^[4]^.

Neutralizing antibodies have become the premier drug developed for infectious diseases for their accessibility from immunized organisms. For SARS-CoV-2, antibodies targeting RBD are more likely to prevent virus entry by blocking the ACE2 receptor or inactivating the spike protein that is an important site for antibody development^[5]^.

The commonly used antibody production methods are hybridoma and phage display. Hybridoma involves immunized animals and humanization, while phage display obtains antibodies with randomly matched heavy and light chain pairs^[6]^. Nowadays, single cell sequencing has accelerated the speed of obtaining antibody sequences by seeking certain memory B cell receptor (BCR) genes directly from infected patients without the introduction of animals or phages^[7–9]^. Thus, antibodies obtained in this way are fully human in origin with paired heavy and light chains. Conversely, along with the shortened time consumption, the selection of sequence candidates becomes harder. A memory B cell gives a theoretical antibody sequence, and numerous results can be obtained.

Molecular modeling (MM) has been used in the development of drug design since the 1950s, which explains and predicts the 3D structure or behavior of molecules^[10]^. By docking with virtual 3D structures generated by computer algorithms, MM could calculate interaction energy to predict how possible a reaction would occur^[11]^. To date, several specialized software or programs have been invented: Rosetta, Schrödinger, Discovery Studio, GROMACS, Autodock, *etc*. MM together with single cell sequencing will fasten the speed of antibody discovery since real world experiments, such as enzyme-linked immunosorbent assay (ELISA), may be abridged. Furthermore, MM is the basis of an AI-based antibody design, which has become a promising method, especially after the emergence of the deep learning algorithm AlphaFold^[12]^.

In this article, single memory B cell BCR sequencing was employed to obtain antibody sequences from a convalescence patient. MM and clustering were performed to select candidate sequences that were further characterized by ELISA, bio-layerinterference (BLI), and pseudovirus neutralization assays. An antibody with broad neutralizing abilities against several SARS-CoV-2 variants, including omicron, was obtained, providing a strategy for antibody development of the emerging infectious disease.

## Materials and methods

### Donors and sera

Twenty-seven donors, who were tested positive for SARS-CoV-2 infection, were treated and recovered three months after the onset between January 24^th^ and April 1^st^, 2020, and one healthy donor was also recruited. The relevant details of each donor are summarized in ***Supplementary Table 1 ***(available online). Eight milliliters of blood were collected from each donor. Sera and peripheral blood mononuclear cells (PBMCs) were isolated and stored at −80 ℃. All procedures were approved by the Ethics Committee of the First People's Hospital of Jiangxia District, Wuhan, China. The study using human blood abided by the principles of the Declaration of Helsinki.

### Cells and virus

293T-ACE2 cells and pseudovirus of SARS-CoV-2 WT, B.1.1.7, B.1.351, P.1, B.1.617.2, and B.1.1.529 were provided by Nanjing Vazyme Biotech Co., Ltd. (Nanjing, Jiangsu, China). 293T-ACE2 cells were cultured at 37 ℃ and 5% CO_2_ with 10% fetal bovine serum in DMEM. HEK 293 freestyle cells were cultured at 37 ℃ and 8% CO_2_ with 120 rpm in FreeStyle 293 expression medium (Cat. #12338018, Gibco, Carlsbad, CA, USA) for antibody expression.

### Serum antibody titers measurement

Spike protein (Cat. #40589-V08B1, SinoBiological, Beijing, China) and nuclear protein (Cat. #40588-V08B, SinoBiological) were coated in 100 ng/well respectively. Five microliters of serum were added with dilution from 1:1000 to 1:12800 followed by a secondary antibody (1:2000 dilution; Cat. #A0293, Sigma-Aldrich, St. Louis, MO, USA). TMB (Cat. #ab171523, Abcam, Cambridge, UK) was added, and the absorbency was measured at a wavelength of 450 nm.

### Single B cell sorting

PBMCs were isolated from sample W25 and resuspended by 40 mL PBS at the amount of 2.24×10^8^ cells followed by B cell enrichment^[13]^ (Cat. #17954, StemCell Technologies, Vancouver, BC, Canada). B cells (8.2×10^6^) were obtained, incubated with spike and RBD proteins (Cat. Nos. 40589-V08B1 [SinoBiological], 40592-V08H [SinoBiological], T80302 [GenScript, Nanjing, Jiangsu, China], and DRA36 [Novoprotein, Suzhou, Jiangsu, China]), stained with CD19-APC-cy7, IgG-BV421 and IgM-BV605 antibodies (Cat. Nos. 561742, 562581 and 562977, Beckman Coulter, Bria, CA, USA) and sorted into a 96 well plate by flow cytometry.

### Single memory B cell BCR full length sequencing

Single memory B cell BCR sequencing was performed by GEXSCOPE sequencing technology^[14]^ (Cat. #4183121, Singleron, Nanjing, Jiangsu, China) with mRNA capture and cDNA reverse-transcription. Two PCR primers were designed for the CH1 region of heavy and light antibody chains by two rounds of semi-nest PCR specific for BCR enrichment products. Specific PCR primers for the library were designed by PCR Handle sequences on the primers of the second semi-nest PCR. Libraries were built, and transcriptome was sequenced followed by BCR mapping and V(D)J gene matching.

### Molecular modeling of the antibodies

Fragment of variable region (Fv) models was created by Antibody Modeling Cascade in Discovery Studio 2019 (DS19), which used an optimized template for homologous modeling^[15]^. The models were then evaluated by the Verify Protein (Profiles-3D) and Ramachandran plot for reliability. Docking was done by inputting the modeled antibodies and RBD structure (7BWJ in PDB)^[16]^ in the ZDOCK program. Two thousand poses were obtained after the ZDOCK program, and then the most suitable one was inputted into the RDOCK program to refine the antigen-antibody complex. The interaction energy of the configuration with the best score in RDOCK was calculated by the Analyze Trajectory program^[17]^.

### Phylogenetic tree of the antibodies

The antibody sequences were clustered by Clustal Omega (https://www.ebi.ac.uk/Tools/msa/clustalo/) and adjusted by V gene distribution and amino acid compositions. A phylogenetic tree of the antibody sequences was generated by ChiPlot (https://xiaochi.chifei3d.com/static/xiaochiPlot/src/index.html).

### Antibody expression and purification

DNA sequences coding the antibody Fv region were synthesized onto the expressing plasmids that contain the constant region of VH and VL by Genscript. VH and VL plasmids were co-transfected into HEK 293 freestyle cells with PEI (Cat. #AC04L092, Life-iLab, Shanghai, China), and the supernatant was purified by the AKTA Pure 25 system with a Hitrap protein A column (Cat. #29048576, Cytiva, Washington, DC, USA).

### RBD binding ELISA assay

RBD (Cat. #CQ201-00, Vazyme, China) was coated at 2 μg/mL. One hundred microliters of expressed antibody was added at the beginning concentration of 2 μg/mL and a half successively for the following wells. Secondary antibody (1:10 000 dilution; Cat. #SA00001-17, Protentech, Chicago, IL, USA) was added followed by TMB and absorbency measurement at a wavelength of 450 nm.

### ACE2 competitive ELISA assay

For sera, the competitive ELISA assay was employed with the Add&Read SARS-CoV-2 S protein RBD/ACE2 Kit (DD2202, Vazyme, China). Four microliters of seurm samples with dilutions from 1:10 to 1:101 920 were added, followed by 4 μL Tag2-ACE2 and Tag1-RBD. The absorbance ratio at 665/620 nm was measured after the involvement of anti-Tag1-Eu and anti-Tag2-A2 antibodies.

For antibodies, the competitive ELISA assay was the same as the RBD binding assay, except for the replacement of 100 μL of expressed antibody by the mixture of 50 μL ACE2 (2 μg/mL, CG205-00, Vazyme) and 50 μL of expressed antibody.

### Affinity measurement

The affinity was measured by BLItz (Octet N1, Sartorius, Göttingen, Niedersachsen, Germany) with Protein G sensors (Cat. #18-5082, Sartorius). The protein G sensors were first soaked in PBS (with 0.02% Tween and 0.1% BSA) for a baseline, loaded with antibodies for 120 s and 30 s in PBS for another baseline. Then, the sensors were soaked in RBD with different concentrations for an association curve and in PBS for a disassociation curve. *K*_on_,* K*_off_, and* K*_d_ were calculated after all runs were finished.

### Epitope mapping

Eleven continuous peptides of RBD (***Supplementary Table 2***, available online) were synthesized by GL Biochem, Shanghai. The assay was the same as the ELISA binding assay except that peptides were coated at a concentration of 10 μg/mL and that the antibody was diluted 10 times successively after a beginning concentration of 10 μg/mL.

### Pseudovirus neutralizing assay

The pseudovirus (Cat. #DD1402, Vazyme) was diluted to 1×10^4^–2×10^4^ TCID_50_/mL and mixed with antibodies of different concentrations. Fifty microliters of 293T-ACE2 cells were added and co-cultured with the virus. Relative light unit (RLU) was measured after 48 h of culture. For the sera, the samples were heated for 30 min at 56 ℃ before the assay.

### Statistical analysis

Serum antibody titers, RBD binding, and RBD-ACE2 inhibition of 27 samples were measured once, while serum neutralization was tested in three replicates and displayed as the mean±SD. Characterization of the antibodies expressed was assessed by ELISA and pseudovirus neutralizing assay in three replicates and displayed as the mean±SD. Affinity was measured with six different concentrations, and *K*_on_, *K*_off_, and *K*_d_ were calculated by Fortebio analysis software of BLItz. Properties of antibody amino acid composition were analyzed according to clusters using one-way ANOVA in graphpad prism 9.0. Half maximal effective concentration (EC_50_) and half maximal inhibitory concentration (IC_50_) were calculated using nonlinear regression, four parameters in graphpad prism 9.0.

## Results

### Characterization of SARS-CoV-2 neutralizing sera

**Figure 1:**
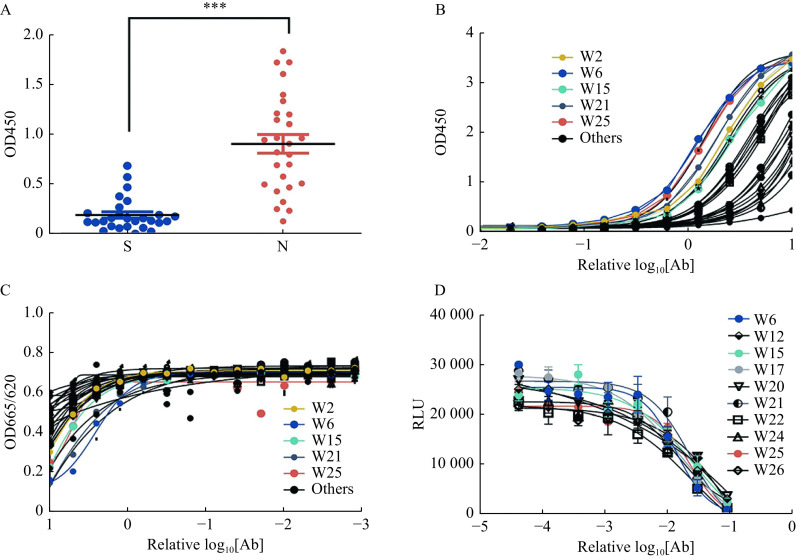
Convalescence serum was able to neutralize SARS-CoV-2.

Blood samples from 27 donors were tested for antibody titer. The titers of antibodies against S protein and nucleoprotein (N) varied a lot: S antibodies and N antibodies could be detected at the diluting ratio of 1:8000 and 1:64000, respectively (***Supplementary Fig. 1***, available online). However, antibody titer showed personal deviation at the diluting ratio of 1:2000 (***Fig. 1A***). ELISA assay found that samples W2, W6, W12, W15, W21, W22, and W25 exhibited a strong binding ability to S protein (***Fig. 1B***), as well as a strong inhibition to RBD-ACE2 interactions (***Fig. 1C***). Some of these samples were chosen for pseudovirus neutralization assay, and the neutralizing ability was detected (***Fig. 1D*** and ***Supplementary Table 1***). Among these samples, W25 had the highest S antibody titer of 1:64000, its relative EC_50_ was 1.423, its inhibition IC_50_ was 5.028 and its neutralization IC_50_ was 0.025, and thus W25 was selected for single memory B cell sequencing.

### Antibody sequences were generated by single memory B cell BCR sequencing

**Figure 2:**
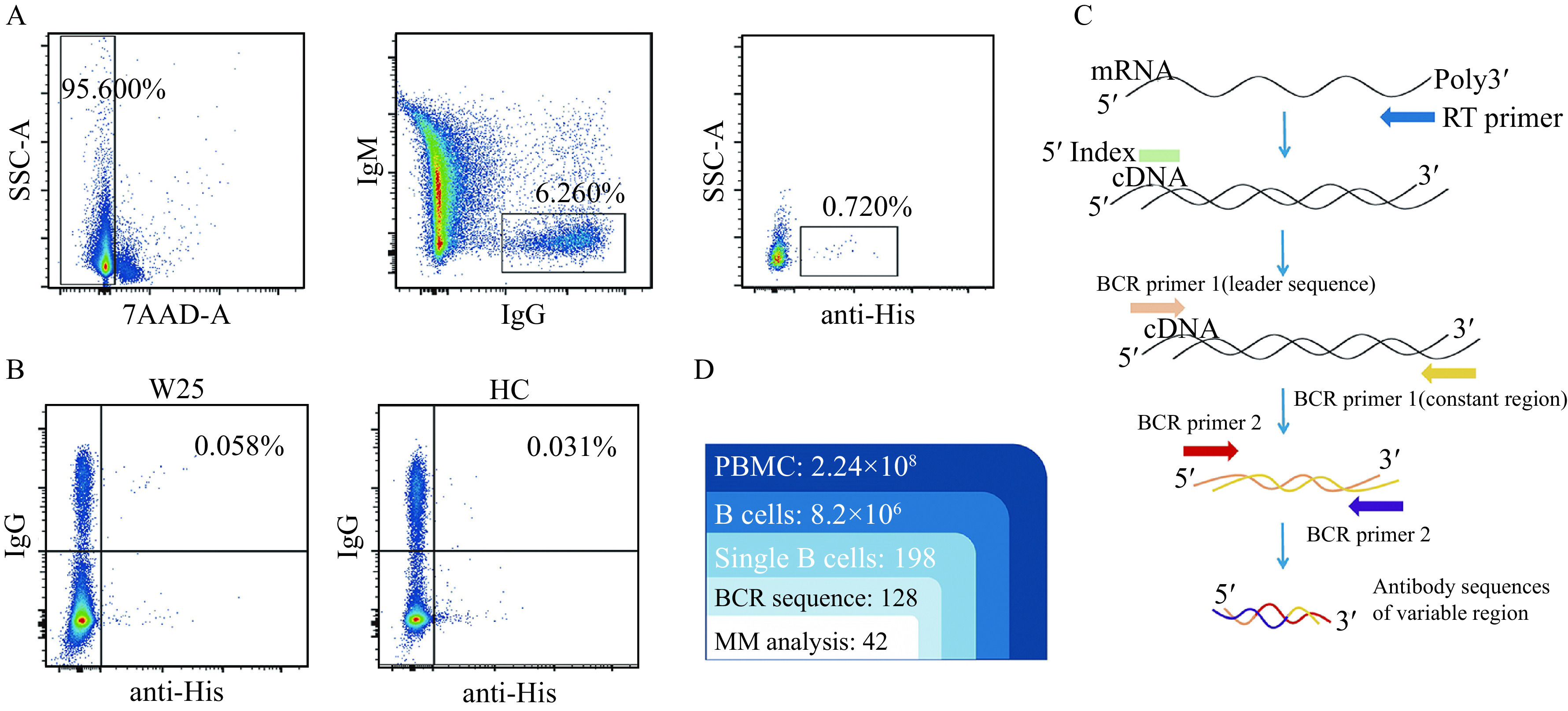
Antibody sequences were generated by single memory B cell BCR sequencing.

PBMCs of W25 were separated, followed by fluorescence activated cell sorting. Spike protein with His-tag was used to pick out S-specific IgG expressing B cells (***Fig. 2A***). The results showed that the radio of His positive cells was significantly different between convalescence and healthy donors (***Fig. 2B***). A total of 198 single B cells were obtained and underwent single memory B cell BCR sequencing, followed by transcribing mRNA into cDNA with additional two rounds of semi-nest PCR using specific primers of both the 5′ and 3′ ends (***Fig. 2C***). A total of 128 sequences were obtained after BCR mapping and packaging from sequence libraries. Forty-two sequences were left by combining highly similar ones and discarding incomplete ones after alignment with the international ImMunoGeneTics information system (IMGT) database (***Fig. 2D***).

### Molecular modeling predicted potential SARS-CoV-2 neutralizing antibodies

The interaction energy given by MM was used to evaluate the potential for antibody neutralization. Here, the ACE2-RBD complex resolved by X-ray^[18]^ was calculated for the interaction energy, resulting in −57.00 kcal/mol that was taken as a standard. Since heavy chain complementarity determining region 3 (HCDR3) played the most important role in an antigen-antibody interaction, it was assumed that only antibodies whose HCDR3-RBD interaction energy was lower or near −57.00 kcal/mol were able to neutralize SARS-CoV-2 by preventing the combination of RBD and ACE2 on host cells.

Homology models of Fv regions were first built for the 42 antibodies and then qualified by the Verify Protein (Profiles-3D) program and Ramachandran plot (***Supplementary Fig. 2***, available online). The results (***Supplementary Table 3***, available online) showed that all the models were theoretically reliable for all scores given by the Profiles-3D program at a higher-than-expected score, and the percentage of amino acids within the credibility interval was higher than 94% in the Ramachandran plot.

**Figure 3:**
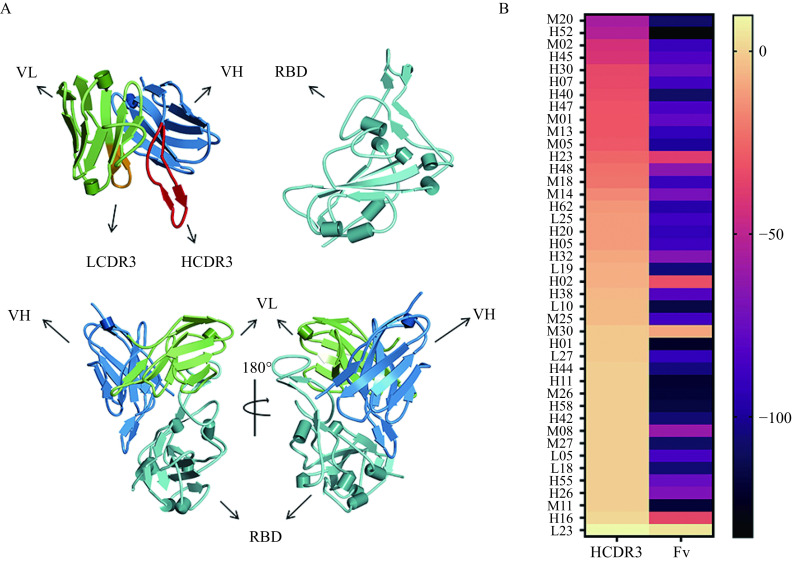
Molecular modeling predicted potential SARS-CoV-2 neutralizing antibodies.

Modeled structures and RBD-antibody complex (7BWJ in PDB)^[16]^ were run in the ZDOCK program, followed by a refinement of the RDOCK program (***Fig. 3A***). The best pose in the RDOCK program was calculated for interaction energy, and only two antibodies had the HCDR3-RBD interaction energy near −57.00 kcal/mol: −53.09 kcal/mol for H52 and −56.49 kcal/mol for M20 (***Fig. 3B***). Besides HCDR3-RBD, the Fv-RBD interaction energy was also calculated, and H52 had the lowest energy of −132.96 kcal/mol.

### Clustering determined candidate sequences to be expressed

**Figure 4:**
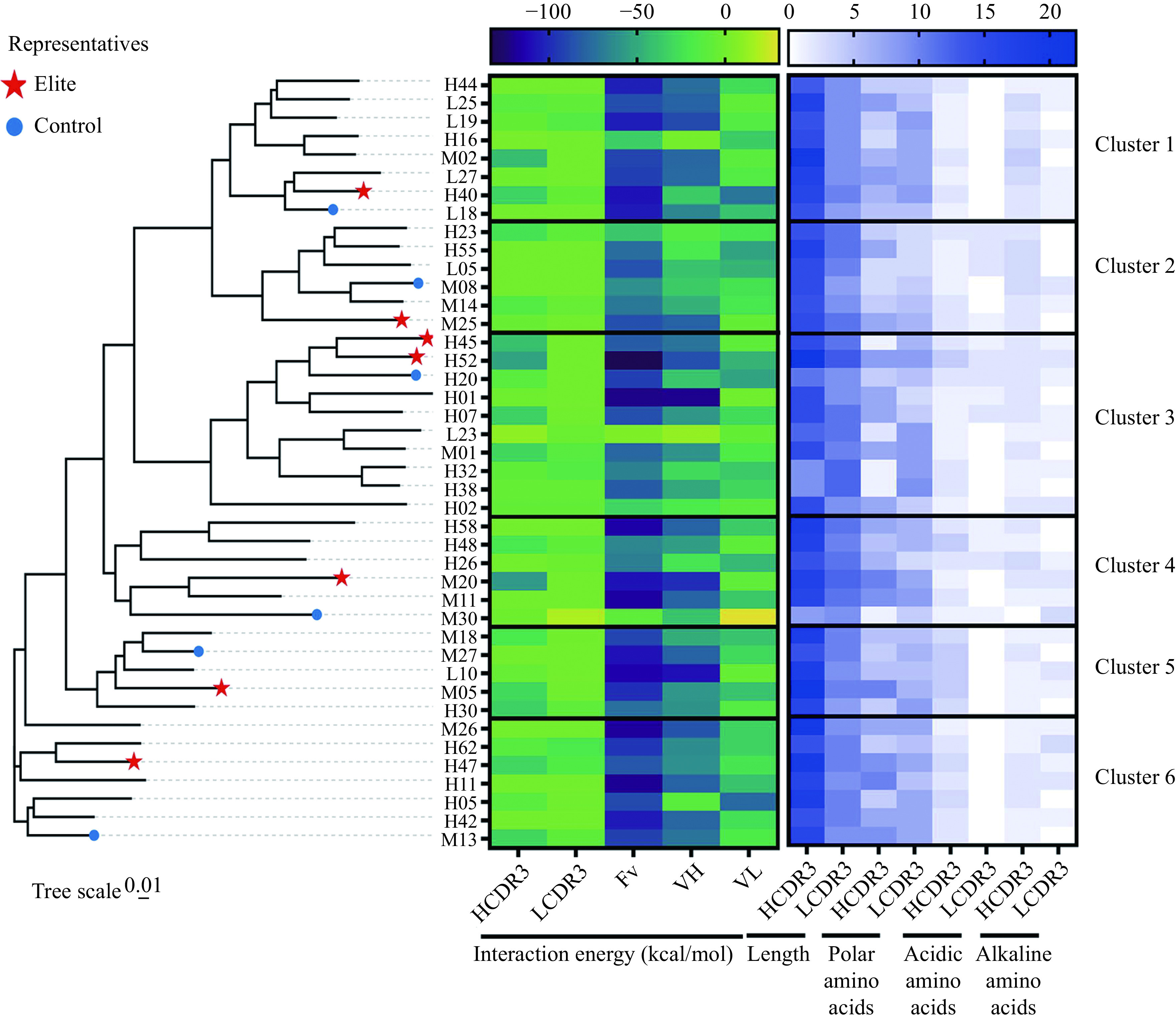
Clustering determined candidate sequences to be expressed.

To cover these sequences as much as possible, some of which were selected as representatives to be expressed and validated with further considerations other than the interaction energy outlined above. The 42 antibodies were clustered into six clusters by their amino acid sequences and optimized with V gene types and MM interaction energy. As CDR3 was of great importance in antigen recognition, the length and amino acid compositions were all analyzed (***Fig. 4***). Among the six clusters, averages of CDR3 length, alkaline, polar and acidic amino acids were calculated. Those with parameters higher than the averages were taken as "elite" antibodies, while those lower than average were taken as control groups. The six clusters had some deviation in their parameters: cluster three had the lowest interaction energy and the fewest polar amino acids (***Supplementary Fig. 3***, available online). Taking all these factors together, seven "elite" and six control antibodies were selected as candidates for the verification (***Supplementary Table 4***, available online).

### Candidate antibodies were verified in real experiments

**Figure 5:**
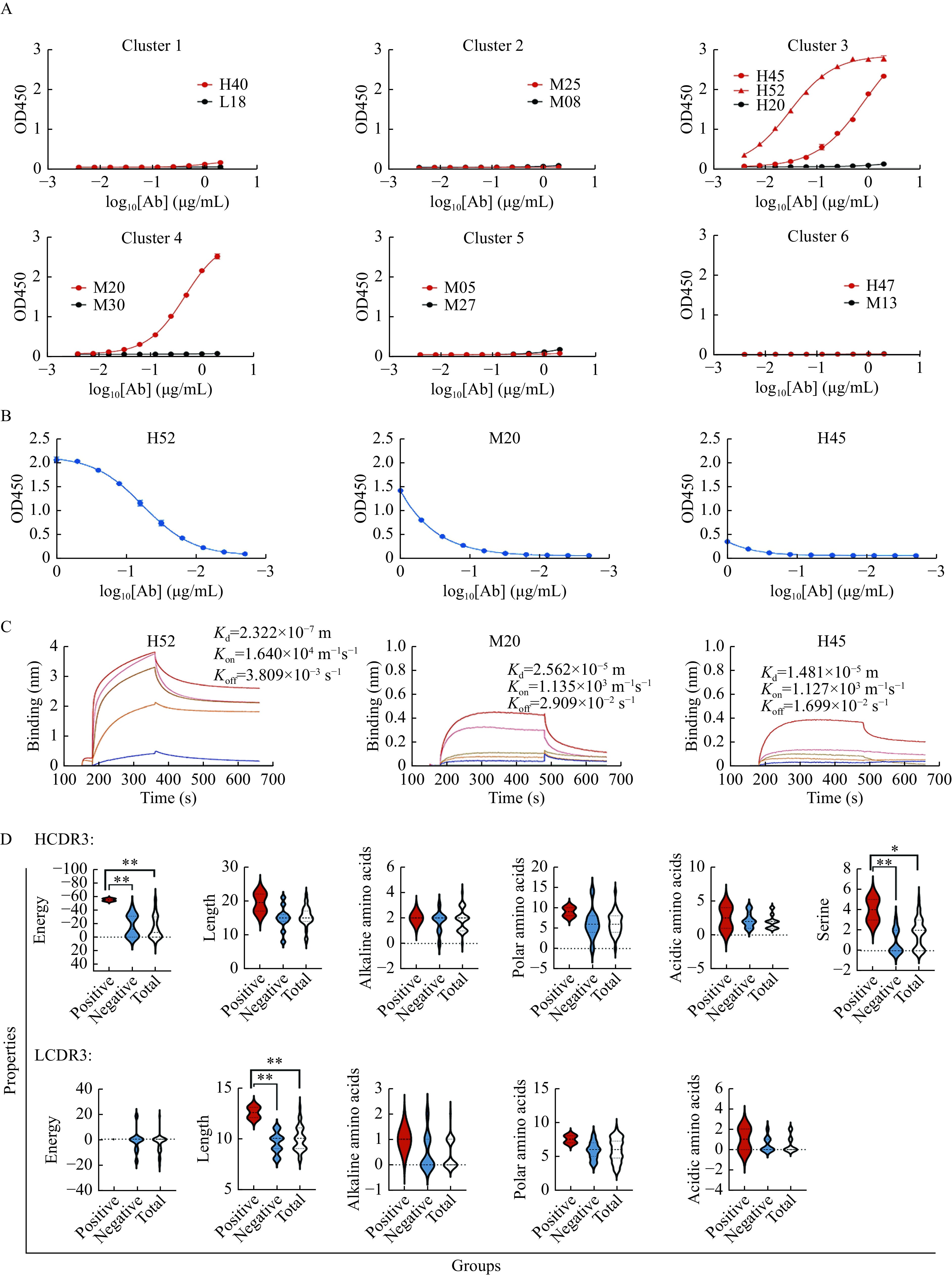
Candidate antibodies were verified in real experiments.

Seven elite and six control antibodies were expressed and purified. RBD recognition was first tested by ELISA, which found that only three antibodies M20, H45 and H52 were positive, while others showed no binding (***Fig. 5A***). The binding ability of M20 and H45 to RBD significantly declined in competitive ELISA with ACE2 as an inhibitor (***Fig. 5B***). As the absorbancy at 450 nm was lower than 0.5 when the concentration of H45 was 1 μg/mL in competitive ELISA, H45 was considered to have no neutralization potential. BLI was used to measure the affinity of the three antibodies. In line with ELISA, H52 showed the best affinity with 10^–7^ mol and 10^–5^ for both M20 and H45, respectively (***Fig. 5C***). Compared with others and the two, M20 and H52 that had neutralization potential, there were longer light chain complementarity determining region 3 (LCDR3) and more serine in HCDR3 (***Fig. 5D***). The properties of these groups were compared using one-way ANOVA in GraphPad Prism 9.0.

### Epitope mapping of antibody H52

**Figure 6:**
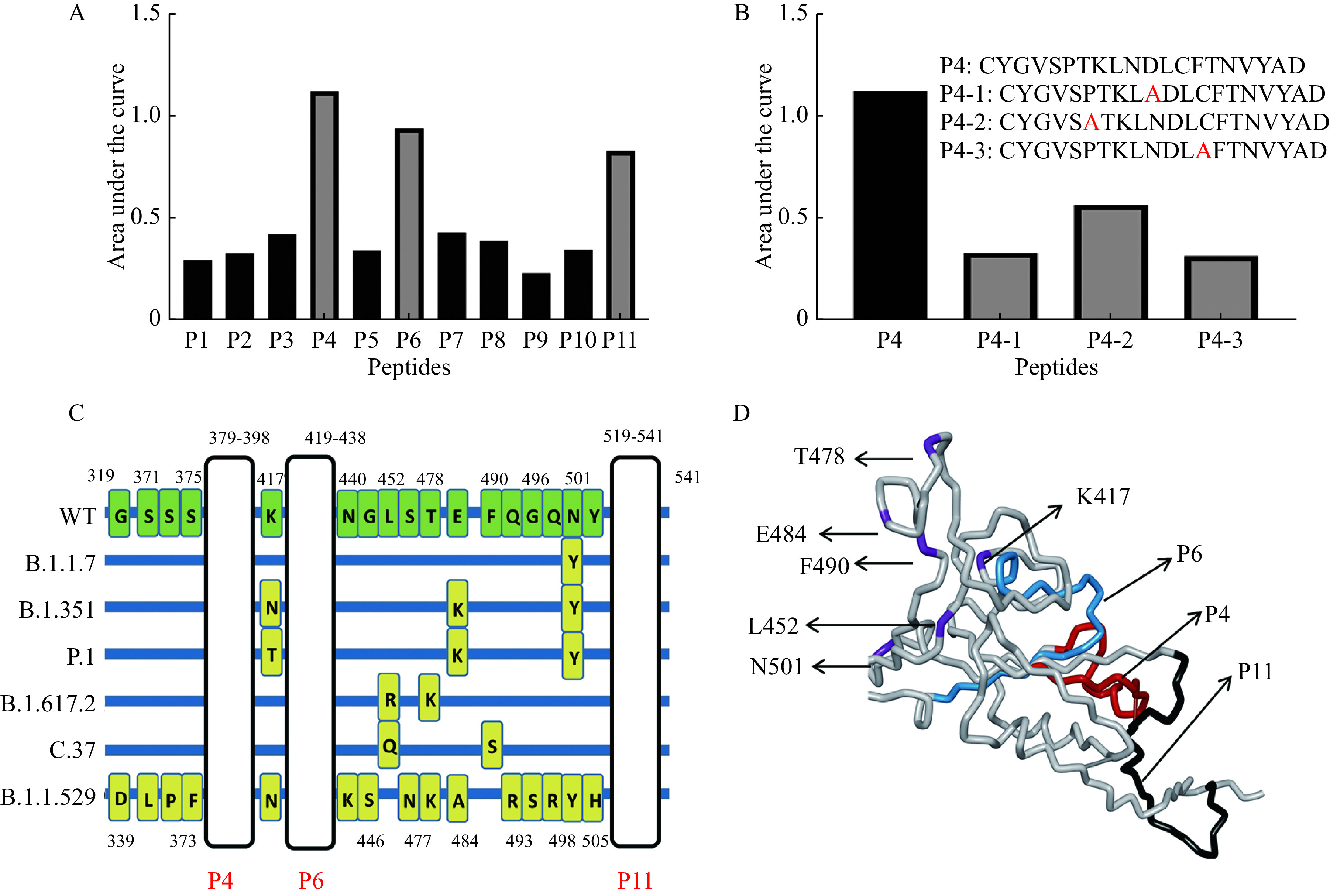
Antibody H52 bound to conserved regions of the receptor binding domain.

RBD was divided into 11 peptides (***Supplementary Table 2***), named P1–P11. Among these peptides, P4, P6, and P11 were confirmed to be the top binding areas by ELISA (***Fig. 6A*** ). Additional three peptides (P4-1, P4-2 and P4-3, ***Supplementary Table 2***) were synthesized with the replacement of certain amino acid to alanine. The binding between P4-1/3 to H52 significantly decreased, indicating that P4 plays an important role in the RBD-H52 interaction (***Fig. 6B***). Mutations in RBD in some important SARS-CoV-2 variants were illustrated, and no mutated sites were found in all three peptides (***Fig. 6C***). The structure of RBD showed that although the three peptides were not adjacent linearly, they were close spatially. Most mutation sites were detected at the interface of RBD-ACE2, which was opposite to peptides P4, P6, and P11 (***Fig. 6D***).

### Antibody H52 was capable of neutralizing several SARS-CoV-2 variants

**Figure 7:**
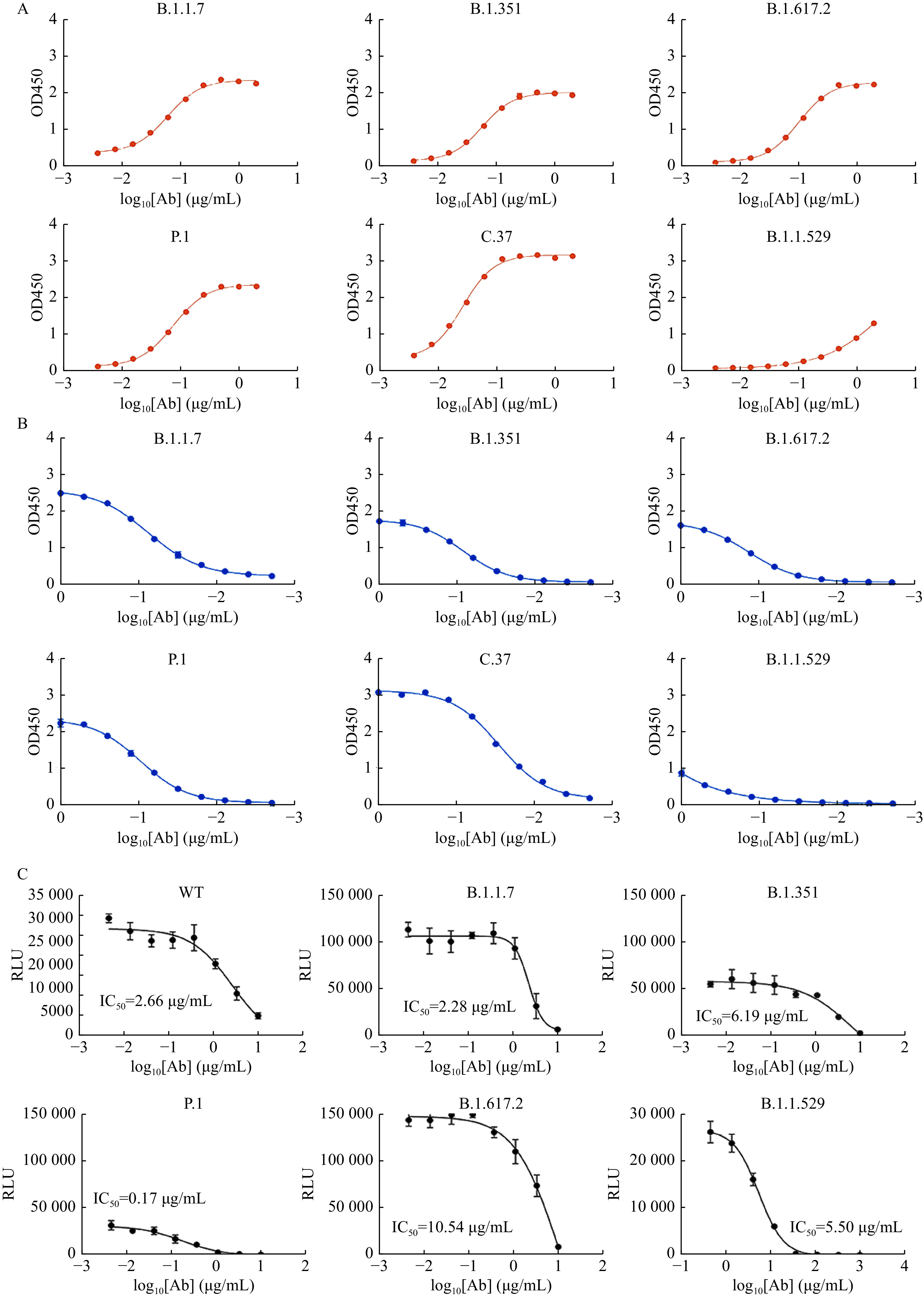
Antibody H52 was capable of neutralizing several SARS-CoV-2 variants.

RBD recognition and ACE2 inhibition were employed, in which H52 was able to bind to and inhibit several SARS-CoV-2 variants with different effects (***Fig. 7A*** and ***B***). Neutralizing ability of H52 was confirmed by the pseudovirus neutralization assay, which showed an IC_50_ of 2.66 μg/mL (***Fig. 7C***). Besides, different protection was discovered against these variants, and the results showed that H52 was a potent neutralizing antibody against variant P.1 with an IC_50_ of 0.17 μg/mL, but had an unsatisfying performance against B.1.617.2 variant with an IC_50_ of 10.54 μg/mL (***Fig. 7C***).

## Discussion

Neutralizing antibodies are important drugs against infectious diseases. For the emerging variants of SARS-CoV-2, more strategies for antibody production are needed.

In the current study, 27 donors who recovered from SARS-CoV-2 infection were recruited. As antibodies underwent a class switch from IgM to IgG with an enhanced humoral immunity^[19]^, the time for sampling was three months after the elimination of the virus to ensure that IgG made up the main subset.

Single cell sequencing accelerates antibody discovery, and thus became the most popular method in the SARS-CoV-2 antibody discovery^[20–23]^. However, a considerable number of cells is needed. The 10× Genomics and BD Rhapsody platform requires 500 and 2000 cells at least, respectively^[24]^. The current study obtained H52 antibody from 198 single memory B cells by GEXSCOPE BCR sequencing with specific primers, which encoded the leading and constant sequence, covered a broad read and yielded a higher rate of output sequences.

The V gene distribution had some features of the 42 antibodies: VH3 occupied the most of all VH genes, which was in line with the common type of B cell repertoire^[25]^, while kappa and lambda light chains were equal in amount, which was different from normal, when kappa is nearly twice the frequency of lambda^[26]^. VK3-20, VK3-15, VK3-11, VK1-5, VK2-30 and VK1-39-01 are the major six V genes of Kappa chain^[27]^ and the dominant one in the current study is VK1-39-01. In addition, besides VK1-39-01, the bias for VH3-30, VH3-53 and VL3-21 was detected in SARS-CoV-2 infected patients^[28]^, which was inconsistent with our antibody H52 (VL1-40, VH1-18) and M20 (VL2-23, VH3-21) due to a lack of samples.

MM predicted potential antibodies for neutralization, especially H52 that was characterized by broad reactivities to many SARS-CoV-2 variants, indicating the desirability of this method for sequence analysis. HCDR3 is in the core of antigen-antibody interaction, and we postulate that only if the interaction energy of HCDR3-antigen is lower or close to that of the antigen-receptor will it be able to prevent their reaction. Moreover, the Fv is where the antibody can recognize its target antigen, so we postulate that the lower the energy is, the more powerful the antibody is, when the reaction of antigen to the receptor can be prevented by the antibody. These assumptions were tested in the current study, and antibody H52, which met the threshold and had the lowest Fv-antigen interaction energy, was the most efficient at neutralizing SARS-CoV-2.

Although MM has been used in predicting antigen-antibody interaction recently^[29]^, its limitations should not be ignored. The major concern about MM is its accuracy— that it does not match experimental results exactly^[30]^, which is caused by a variety of factors: inadequate templates for modeling^[31]^, conformational change in the actual reaction^[32]^ and inaccuracies in the dock algorithm that calculates intricate macromolecules systems^[33]^. In the curretn study, the MM prediction was based on blocking of ACE2-RBD interactions, which was only one of neutralizing mechanisms. Antibodies that employed other mechanisms, such as steric hindrance or conformational disruption, may not be selected in this way, indicating the existence of false negative errors. As a newly emerged method involved in multi-subjects, MM still needs to be improved for better performance.

Similar sequences have similar structures and possess similar functions. For antibodies, similar sequences may recognize a certain kind of epitope, which makes clustering a reasonable way to narrow down candidates from numerous sequences. Another advantage of the clustering analysis is that if one representative of a cluster behaves excellently, this cluster will be of value for a detailed study, by which better ones would be discovered or sequence information for functional improvement would be found. In the current study, seven "elite" antibodies from six clusters were tested in ELISA, but only three of them recognized RBD, the rest might recognize non-RBD regions (NTD, or S2 subunit) or even resulted from mis-captured B cells. These clusters do have some but not statistically significant deviaion, which illustrates the complexity of antibody sequences.

The length of HCDR3 and the framework pairing are regarded as factors for antibody function^[34–35]^. In the six clusters, we selected control sequences with factors higher or lower than the average to investigate key elements in SARS-CoV-2 antibodies. The two antibodies, H52 and M20, showed a better inhibition, which was in line with MM prediction, and exhibited some common characteristics: longer LCDR3 and more serine in HCDR3, which might provide some reference for SARS-CoV-2 antibody selection.

SARS-CoV-2 invades host cells using RBD. Studies revealed that within its spike protein and structural amino acids, A475, N487, E484, Y453 and N501 play important roles in the interaction between RBD, virus and ACE2 in host cells^[18]^. Several variants of concern (VOCs), such as B.1.1.7, B.1.351, P.1, B.1.617.2, and B.1.1.529 as proposed by the WHO, possess mutations around those crucial amino acids mentioned above, enhancing virus spreading and evasion^[36–39]^. Antibody H52 here revealed a strong binding to P4 (379–398), P6 (419–438), and P11 (519–541). These areas were away from the RBD-ACE2 interface and had few strands or loops around, providing possibilities for direct antibody contact. The three peptides are not adjacent linearly but close spatially, indicating a conformational epitope of antibody H52. More importantly, the peptides have no mutations among VOCs, suggesting the conservativeness of these areas and that the evolution of SARS-CoV-2 virus is mainly to strengthen RBD-ACE2 interaction. Furthermore, the latest variants of SARS-CoV-2, including omicron and its lineage BA.1, BA.2, BA.3^[40]^ and their recombinant omicron XE, XD and XF, retained conservativeness on peptide P4, P6, and P11. The antibody H52 may retain broad neutralization for these variants and remain to be tested in future experiments.

The neutralization ability of H52 is not satisfactory enough to fight against the virus, which may just be the result of indirect inhibition by steric hindrance. On the other hand, an indirect inhibition gives rise to a reduced chance of escape, or perhaps the limited neutralization may be enhanced by adding other potent antibodies for cocktail therapies. Moreover, the conserved region P4, P6 and P11 may be used as the target for more powerful antibodies or vaccine production rather than the RBD-ACE2 interface that mutates fast.

With the emergence of SARS-CoV-2 mutations, several neutralizing antibodies have displayed declined protection, which is called antibody resistance^[41]^. It is of great significance to develop more powerful antibodies, but this is hard for traditional methods in a limited time. However, single cell sequencing generates numerous antibody sequences, and MM helps select interested ones. For the virus variants, the mutated RBD protein can be uploaded as a template to select antibodies with a high reactivity *in silico* and thus decrease the time spent, which is helpful to avoid antibody resistance.

In the current study, we employed single memory B cell BCR sequencing from a donor with a high S protein antibody titer and neutralization. Forty-two sequences from sequence libraries were selected for MM and clustering analysis. Thirteen antibodies were expressed, three of which were tested positive for RBD recognition, and one exhibited a broad neutralization. These results have provided a strategy for screening antibodies from numerous sequences, which is able to expedite the antibody research period. Additionally, we propose that peptides P4, P6, and P11 are conserved regions of SARS-CoV-2 RBD, and may be a target for better antibody and vaccine design.

## Fundings

This study was supported by the Jiangsu Provincial Key Research and Development Program (Grant No. BE2020616), the National Key R&D Program of China (Grant No. 2018YFC1200603), the National Science and Technology Major Project (Grant No. 2019SWAQ05-5-4) and Jiangsu Key Lab of Cancer Biomarkers, Prevention and Treatment, Collaborative Innovation Center for Cancer Personalized Medicine, Nanjing Medical University.

## SUPPLEMENTARY DATA

Supplementary data to this article can be found online.
