# Supplementary material for: A SARS-CoV-2 neutralizing antibody discovery by single cell sequencing and molecular modeling
Source: J Biomed Res. 2022 Dec 12;37(3):166–78. doi: 10.7555/JBR.36.20220221 (PMC10226085; doi:10.7555/JBR.36.20220221)
Supplement: Supplementary file 1 — Supplementary data to this article can be found online. [file jbr-37-166-S1.pdf]

Available online at www.jbr-pub.org.cn

Open Access at PubMed Central

The Journal of Biomedical Research, 2022 37(0): 1–13

Supplementary Data

JBR

A SARS-CoV-2 neutralizing antibody discovery by single cell sequencing and molecular modeling

Zheyue Wang[1,△], Qi Tang[1,△], Bende Liu[2,△], Wenqing Zhang[1], Yufeng Chen[1], Ningfei Ji[3], Yan Peng[1], Xiaohui Yang[1], Daixun Cui[1], Weiyu Kong[1], Xiaojun Tang[4], Tingting Yang[1], Mingshun Zhang[1], Xinxia Chang[1], Jin Zhu[5,✉], Mao Huang[3,✉], Zhenqing Feng[1,6,✉]

[1]*National Health Commission Key Laboratory of Antibody Technique, Jiangsu Province Engineering Research Center of Antibody Drug, Department of Pathology, Nanjing Medical University, Nanjing, Jiangsu 211166, China;*
[2]*Department of Cardiology, the First People's Hospital of Jiangxia District, Wuhan, Hubei 430299, China;*
[3]*Department of Respiratory and Critical Care Medicine, the First Affiliated Hospital of Nanjing Medical University, Nanjing, Jiangsu 210029, China;*
[4]*Department of Rheumatology and Immunology, the Affiliated Drum Tower Hospital of Nanjing University Medical School, Nanjing, Jiangsu 210008, China;*
[5]*Huadong Medical Institute of Biotechniques, Nanjing, Jiangsu 210028, China;*
[6]*Jiangsu Key Lab of Cancer Biomarkers, Prevention and Treatment, Collaborative Innovation Center for Cancer Personalized Medicine, Nanjing Medical University, Nanjing, Jiangsu 211166, China.*

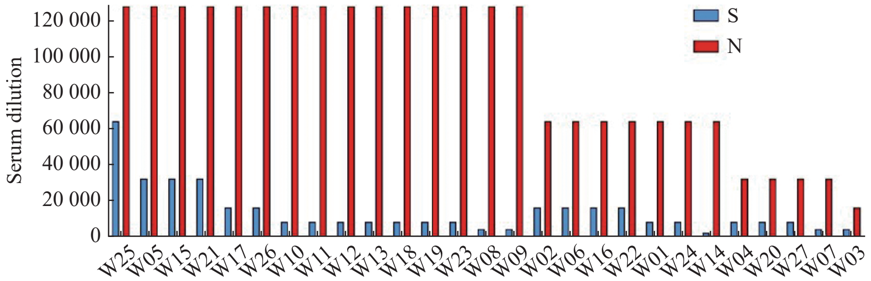

Supplementary Fig. 1 **Antibody titers of S and N proteins for 27 donors.** Sera titers for the main antigen of SARS-CoV-2, spike protein (S) and nucleoprotein (N) of the 27 convalescence patients were examined by enzyme-linkedimmunosorbent assay, related to ***Fig. 1***.

△These authors contributed equally to this work.

✉Corresponding authors: Jin Zhu, Huadong Medical Institute of Biotechniques, 293 Zhongshan Road, Nanjing, Jiangsu 210028, China. Tel: +86-25-84514223, E-mail: zhujin1968@njmu.edu.cn; Mao Huang, Department of Respiratory and Critical Care Medicine, the First Affiliated Hospital of Nanjing Medical University, 300 Guangzhou Road, Nanjing, Jiangsu 210029, China. Tel: +86-25-83718836, E-mail: hm6114@163.com; Zhenqing Feng, National Health Commission Key Laboratory of Antibody Technique, Jiangsu Province Engineering Research Center of Antibody Drug, Department of Pathology, Nanjing Medical University, 101 Longmian Ave., Nanjing, Jiangsu 211166, China. Tel: +86-25-86869411, E-mail: fengzhenqing@njmu.edu.cn.

Received: 05 October 2022; Revised: 06 December 2022; Published online: 12 December 2022

CLC number: R392, Document code: A

The authors reported no conflict of interests.

This is an open access article under the Creative Commons Attribution (CC BY 4.0) license, which permits others to distribute, remix, adapt and build upon this work, for commercial use, provided the original work is properly cited.

© 2022 by the Journal of Biomedical Research.

https://doi.org/10.7555/JBR.36.20220221

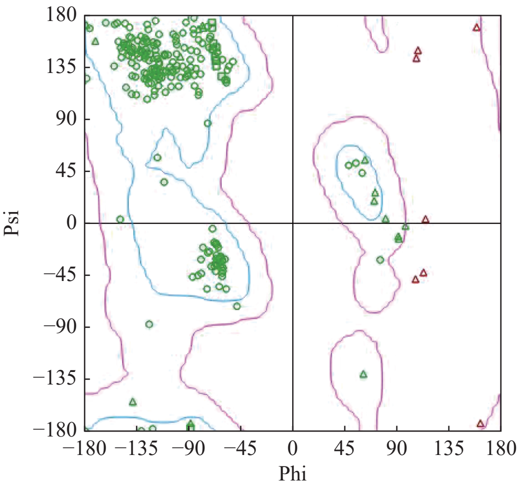

Supplementary Fig. 2 **Ramachandran plot of modeled antibodies, H52 as an example.** Ramachandran plot evaluates structural stability by calculating amino acid numbers in credibility interval. Each point within the plot was a single amino acid, and the ones within blue lines were believed to be reliable while those out of rosy lines were unreliable. A model with 90% of amino acids in the blue lines is credible. The percentages of the 42 antibody models were calculated and summarized in ***Supplementary Table 2***.

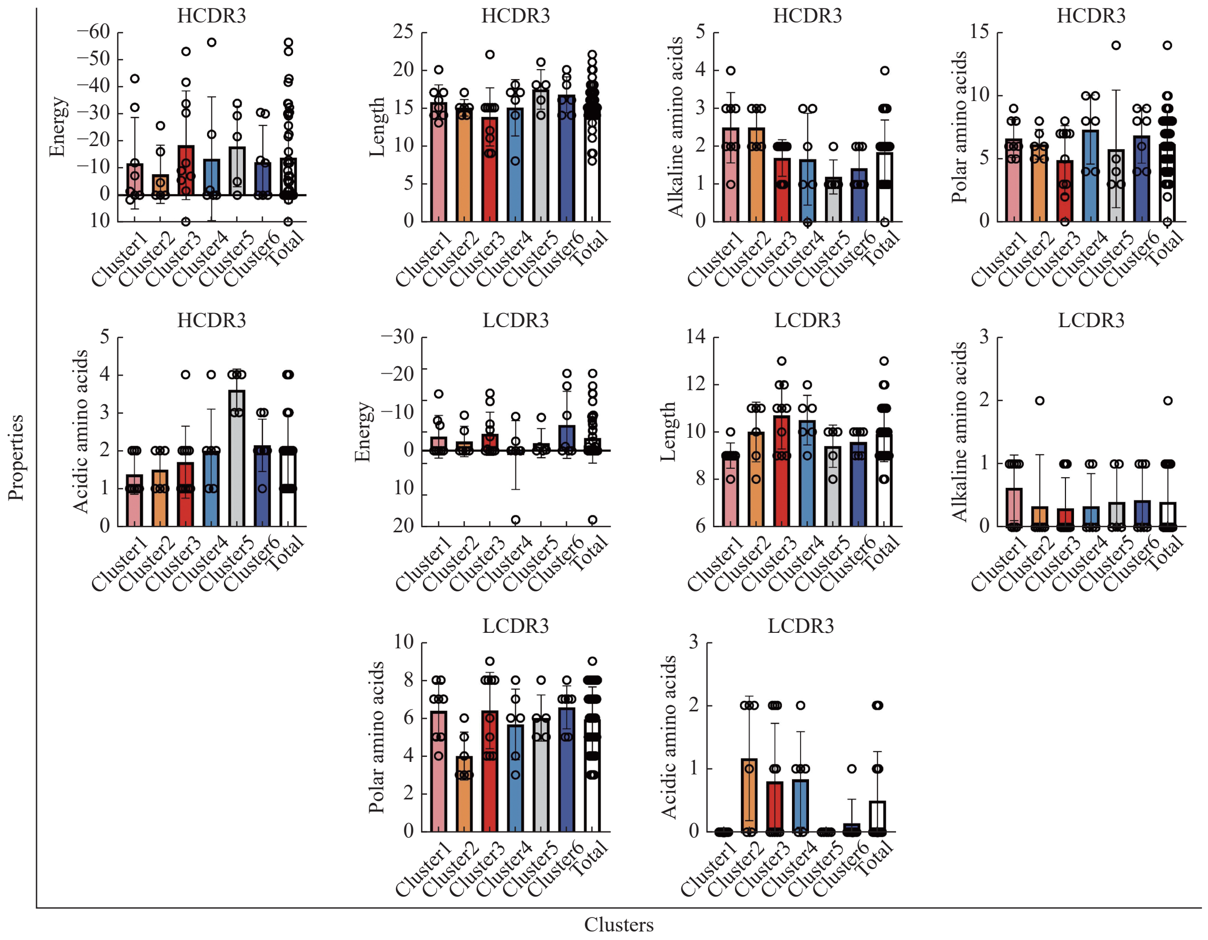

Supplementary Fig. 3 **Properties of six clusters.** Properties of the 42 antibodies, six clusters were analyzed on HCDR3 and LCDR3. The properties included interaction energy, which was calculated by molecular modeling, length and amino acid composition of the CDR, related to figure 4. Data are shown as mean±SD, n=42. Statistic analysis of antibody properties was performed using one-way ANOVA. HCDR3: heavy chain complementarity determining region 3; LCDR3: light chain complementarity determining region 3.

Supplementary Table 1 **Serum validation of 27 convalescence donors**

			Serum				
Sample	Gender	Age (years)	Titer for S	Titer for N	Binding EC_{50}	Inhibition IC_{50}	Neutralizing IC_{50}
W1	Female	30	8000	64 000	4.238	27 878	na
W2	Female	29	16 000	64 000	2.385	12.91	na
W3	Female	44	4000	16 000	6771	5.642	na
W4	Female	40	8000	32 000	8.271	26 895 008 978	na
W5	Female	40	32 000	128 000	8095	6.946	na
W6	Male	57	16 000	64 000	1.243	2.589	0.015 11
W7	Male	6	4000	32 000	9.987	5.124	na
W8	Female	27	4000	128 000	7827	10.66	na
W9	Female	46	4000	128 000	7510	2.215	na
W10	Female	39	8000	128 000	5.84	21.51	na
W11	Male	33	8000	128 000	4848	6.361	na
W12	Female	55	8000	128 000	2.567	9.124	24 916
W13	Male	58	8000	128 000	5.677	8.439	na
W14	Female	38	2000	64 000	13.5	4.839	na
W15	Female	24	32 000	128 000	2.756	15.39	0.028 78
W16	Female	36	16 000	64 000	6.271	68 386	na
W17	Female	30	16 000	128 000	3.802	150 933	0.018 28
W18	Female	26	8000	128 000	3.91	175 381	na
W19	Female	25	8000	128 000	23.51	18 537 391	na
W20	Male	25	8000	32 000	7.687	1744	7.34177E+12
W21	Female	27	32 000	128 000	1.953	18.09	0.025 87
W22	Female	26	16 000	64 000	1.42	444.5	0.027 41
W23	Female	24	8000	128 000	31 299	663.6	na
W24	Female	24	8000	64 000	2.376	2152	0.015 81
W25	Female	41	64 000	128 000	1.423	5.028	0.024 81
W26	Female	30	16 000	128 000	3.746	344.1	0.252 2
W27	Female	34	8000	32 000	12 073	54.49	na
Average		33.85	10 079.37	80 634.95			

S: spike protein; N: nucleoprotein; EC_{50}: half maximal effective concentration; IC_{50}: half maximal inhibitory concentration. na: not available.

Supplementary Table 2 **Receptor binding domain peptide sequences**

Peptide ID	Amino acid sequence	Peptide ID	Amino acid sequence
P1	RVQPTESIVRFPNITNLCPF	P8	SNLKPFERDISTEIYQAGST
P2	GEVFNATRFASVYAWNRKRI	P9	PCNGVEGFNCYFPLQSYGFQ
P3	SNCVADYSVLYNSASFSTFK	P10	PTNGVGYQPYRVVVLSFELL
P4	CYGVSPTKLNDLCFTNVYAD	P11	HAPATVCGPKKSTNLVKNKCVNF
P5	SFVIRGDEVRQIAPGQTGKI	P4-1	CYGVSPTKLADLCFTNVYAD
P6	ADYNYKLPDDFTGCVIAWNS	P4-2	CYGVSATKLNDLCFTNVYAD
P7	NNLDSKVGGNYNYLYRLFRK	P4-3	CYGVSPTKLNDLAFTNVYAD

Supplementary Table 3 **Amino acid sequences of 42 antibodies and holomogy modeling assessment**

	Sequence information						Verify Protein (Profiles-3D)			Ramachandran plot		
ID	VH	IGHV gene	IGHJ gene	VL	IGLV gene	IGLJ gene	Veried score	Ex high	Ex low	Total amino acids	Unbelievable amino acids	Credibility (%)
H40	QVQLQESGPGLVKPSETLSLTCTVSGGSVSSGGYYWSWIRQPPGKGLEWIGYIYYSGSTNYNPSLKSRVTISVDTSKNQFSLKLSSVTAADTAVYYCAREALSPTSHTSGMDVWGQGTTVTVSS	VH4-61	JH3	EIVLTQSPGTLSLSPGERATLSCRASQSVSSSYLAWYQQKPGQAPRLLIYGASSRATGIPDRFSGSGSGTDFTLTISRLEPEDFAVYYCQQYGSSPLYTFGQGTKLEIK	VK3-20	JK2	122.58	105.81	47.61	233	10	95.71
L27	QLQLQESGPGLVKPSETLSLTCTVSGGSISSSSYYWGWIRRGKKKKEWIGSIYYSGSTYYNPSLKSRVTISVDTSKNQFSLKLSSVTAADTAVYYCARQISSTSLTSKRYFDLWGRGTLVTVSS	VH4-39	JH2	EIVLTQSPGTLSLSPGERATLSCRASQSVSSSYLAWYQQKPGQAPRLLIYGASSRATGIPDRFSGSGSGTDFTLTISRLEPVDFAVYYCQQSGGSPRTFGQGTKVEVK	VK3-20	JK1	95.03	93.46	45.06	206	10	95.15
L18	QLQLQESGPGLVKPSETLSLTCTVSGGSISSSSYYWGWIRQPPGKGLEWIGSIYYSGSTYYNPSLKSRVTISVDTSKNQFSLKLSSVTAADTAVYYCARHAGGSSWYPFDYWGQGTLVTVSS	VH4-39	JH4	EIVLTQSPATLSLSPGERATLSCRASQSVSSYLAWYQQKPGQAPRLLIYDASNRATGIPARFSGSGSGTDFTLTISSLEPEDFAVYYCQQRSNWPTFGGGTKVEIK	VK3-11	JK4	130.59	103.52	46.58	228	5	97.81
M02	QVQLQQWGAGLLKPSETLSLTCAVYGGSFSGYYWSWIRQPPGKGLEWIGEINHSGSTNYNPSLKSRVTISVDTSKNQFSLKLSSVTAADTAVYYCARVELLRRGYSYGNKGSFDYWGQGTLVTVSS	VH4-34	JH4	DIQMTQSPSSLSASVGDRVTITCRASQSISSYLNWYQQKPGKAPKLLIYAASSLQSGVPSRFSGSGSGTDFTLTISSLRPEDFATYYCQQSFNTPQTFGQGTKLEIK	VK1-39-01	JK2	109.5	105.81	47.61	233	10	95.71
H16	QVQLQQWGAGLLKPSETLSLTCAVYGGSFSGYYWSWIRQPPGKGLEWIGGNRDSGSTNYNPSLKSRVTISVDTSKNQFSLKLSSVTAADTAVYYCARGSSIAAAGRRLDYWGQGTLVTVSS	VH4-34	JH4	DIQMTQSPSSLSASVGDRVTITCRASQSISSYLNWYQQKPGKAPKLLRDAASSLQSGVPSRFSGSGSGTDFTLTISSLQPEDFATYYCQQSYSTPRTFGQGTKVEIK	VK1-39-01	JK1	113.6	103.52	46.58	228	8	96.49

Supplementary Table 3 **Amino acid sequences of 42 antibodies and holomogy modeling assessment** (continued)

	Sequence information						Verify protein (profiles 3D)			Ramachandran plot		
ID	VH	IGHV gene	IGHJ gene	VL	IGLV gene	IGLJ gene	Veried score	Ex high	Ex low	Total amino acids	Unbelievable amino acids	Credibility (%)
H44	QLQLQESGPGLVKPSETL<br>SLTCTVSGGSISSSSYYW<br>GWIRQPPGKGLEWIGSIY<br>YSGSTYYNPSLKSRVTIS<br>VDTSNNEFSLRLRSVTAA<br>DTAVYYCAKLGSGTEN<br>WFDPWGQGTLVTVSS	VH4-39	JH5	DIQMTQSPSSVSASAGDR<br>VTITCRASQGISSWLAWY<br>QQKPGKAPKLLIYAASSL<br>QSGVPSRFSGVRVWGDF<br>TLTISSLQPEDFATYYCQ<br>QAKSFPLTFGGGTKVEIK	VK1-12	JK4	121.76	103.52	46.58	228	9	96.05
L19	QLQLQESGPGLVTPSETL<br>SLTCTVSGGSISSSSYYW<br>GWIRQPPGKGLEWIGSIY<br>YSGSTYYNPSLKSRVTIS<br>VDTSKNQFSLKLSSVTAA<br>DTAVYYCARHAGGSSW<br>YPFDYWGQGTLVTVSS	VH4-39	JH4	AIRMTQSPSSFSVSIGDRV<br>TITCRASQGISSYLAWYQ<br>QKPGKAPKLLIYAASTLQ<br>SGVPSRFSGSGSGTDFTL<br>TISSLQSEDFATYYCQQY<br>YTYPYTFGQGTKLEIK	VK1-8	JK2	121.77	103.98	46.79	229	7	96.94
L25	LLVTPSETLSLTCTVSGG<br>SISSSSYYWGWIRQPPGK<br>GLEWIGSIYYSGSTYYNP<br>SLKSRVTISVDTSKNQFS<br>LKLSSVTAADTAVYYCA<br>RQISSTSLTSKRYFDLWG<br>RGTLVTVSS	VH4-39	JH2	DIQMTQSPSSLSASVGDR<br>VTITCRASQGISNYLAWY<br>QQKPGKVPKLLIYAASTL<br>QSGVPSRFSGSGSGTDFT<br>LTISSLQPEDVATYYCQK<br>YNSAPLTFGGGTKVEIK	VK1-27	JK4	111.99	100.78	45.35	222	7	96.85
M27	EVQLLESGGGLVQPGGS<br>LRLSCAASGFTFSSYAMS<br>WVRQAPGKGLEWVSAIS<br>GSGGSTYYADSVKGRFTI<br>SRDNSKNTLYLQMNSLR<br>AEDTAVYYCAKDRYVD<br>TAMDFDYWGQGTLVTV<br>SS	VH3-23	JH4	EIVMTQSPATLSVSPGER<br>ATLSCRASQSVSSNLAW<br>YQQKPGQAPRLLIYGAST<br>RATGIPARFSGSGSGTEG<br>TLTISSLQSEDFAVYYCQ<br>QYNNWPPWTFGQGTKV<br>EIK	VK3-15	JK1	117.29	103.98	46.79	229	5	97.82
M26	QVQLVESGGGVVQPGRS<br>LRLSCAASGFTFSSYGMH<br>WVRQAPGKGLEWVAVIS<br>YDGSNKYYADSVKGRFT<br>ISRDNSKNTLYLQMNSL<br>KSEDTAVYFCARVSQAN<br>SNAIMIPAAIFDSWGQGT<br>LVTVSS	VH3-30	JH5	DIQMTQSPSTLSASVGDR<br>VTITCRASQGISNYLAWF<br>QQKPGKAPKSLIYAASSL<br>QSGVPSKFSGSGSGTDFT<br>LTISSLQPEDFATYYCQQ<br>YRSYPYTFGQGTNLEIK	VK1-16	JK2	121.14	106.27	47.82	234	8	96.58

Supplementary Table 3 **Amino acid sequences of 42 antibodies and holomogy modeling assessment** (continued)

	Sequence information						Verify protein (profiles 3D)			Ramachandran plot		
ID	VH	IGHV gene	IGHJ gene	VL	IGLV gene	IGLJ gene	Veried score	Ex high	Ex low	Total amino acids	Unbelievable amino acids	Credibility (%)
H11	EVQLVESGGGLVQPGGS LRLSCAASGFTFSDHYM DWVRQAPGKGLEWVGR TRNKANSYTTEYAASVK GRFTISRDDSKNSLYLQM NSLKTEDTAVYYCARSS TYSRDYYYYMDVWGKG TTVTVSS	VH3-72	JH6	DIQMTQSPSAMSASVGD RVTITCRASQGIRNDLGW YQQKPGKAPKRLIYAAS SLQSGVPSRFSGSGSGTE FTLTISSLQPEDFATYYCL QHNSYPLTFGGGTKVEIK	VK1-17	JK4	123.89	105.35	47.41	232	12	94.83
H30	EVQLVESGGGLVKPGGS LRLSCAASGFTFSSYSMN WVRQAPGKGLEWVSSIS SSSSYIYYADSVKGRFTIS RDNAKNSLYLQMNSLRA EDTAVYYCARSELLWFG EWYAFDIWGQGTMVTV SS	VH3-21	JH3	EIVLTQSPGTLSLSPGERA TLSCRASQSVSSSYLAW YQQKPGQAPRLLIYGASS RATGIPDRFSGSGSGTDF TLTISRLEPEDFAVYYCQ QYGSSYTFGQGTKLEIK	VK3-20	JK2	116.61	104.44	47.00	230	6	97.39
M13	QVQLVESGGGLVKPGGS LRLSCAASGFTFSDYYMS WIRQAPGKGLEWVSYISS SGSTIYYADSVKGRFTIS RDNAKNSLYLQMNSLRA EDTAVYYCARGLNWNY DYYYYMDVWGKGTTVT VSS	VH3-11	JH6	DIQMTQSPSSLSASVGDR VTITCRASQSISSYLNWY QQKPGKAPKLLIYAASSL QSGVPSRFSGSGSGTDFT LTISSLQPEDFATYYCQQ SYSTPWTFGQGTKVEIK	VK1-39-01	JK1	122.68	104.44	47.00	230	5	97.83
H05	EVQLVESGGGLVQPGGS LRLSCAASGFTFSSYDMH WVRQATGKGLEWVSAI GTAGDTYYPGSVKGRFTI SRENAKNSLYLQMNSLR AGDTAVYYCARAVHSSE VLPGENWFDPWGQGTL VTVSS	VH3-13	JH5	DIQMTQSPSSLSASVGDR VTITCRASQSISSYLNWY QQKPGKAPKLLIYAASSL QSGVPSRFSGSASGTDFT LTISSLQPEDFATYFCQQS YGTPPWTFGQGTKVEIK	VK1-39-01	JK1	115.32	105.35	47.41	232	12	94.83
H42	EVQLVESGGGLIQPGGSL RLSCAASGFTVSSNYMS WVRQAPGKGLEWVSVIY SGGSTYYADSVKGRFTIS RDNSKNTLYLQMNSLRA EDTAVYYCARETYGSGS YYFDYWGQGTLVTVSS	VH3-53	JH4	DIQMTQSPSSLSASVGDR VTITCRASQSISSYLNWY QQKPGKAPKLLIYAASSL QRGVPSRFSGSGSGTDFT LTISSLQPEDFATYYCQQ SYSTPQLTFGGGTKVEIK	VK1-39-01	JK4	114.61	103.52	46.58	228	10	95.61

Supplementary Table 3 **Amino acid sequences of 42 antibodies and holomogy modeling assessment** (continued)

	Sequence information						Verify protein (profiles 3D)			Ramachandran plot		
ID	VH	IGHV gene	IGHJ gene	VL	IGLV gene	IGLJ gene	Veried score	Ex high	Ex low	Total amino acids	Unbelievable amino acids	Credibility (%)
H47	EVQLVESGGVVVQPGGS<br>LRLSCAASGFTFDDYTM<br>HWVRQAPGKGLEWVSLI<br>SWDGGSTYYADSVKGRF<br>TISRDNSKNSLYLQMNSL<br>RTEDTALYYCAKDTVDT<br>AMGHYYYYYMDVWGK<br>GTTVTVSS	VH3-43	JH6	AIRMTQSPSSFSASTGDR<br>VTITCRASQGISSYLAWY<br>QQKPGKAPKLLIYAASTL<br>QSGVPSRFSGSGSGTDFT<br>LTISCLQSEDFATYYCQQ<br>YYSYPPLTFGGGTKVEIK	VK1-8	JK4	114.53	106.27	47.82	234	7	97.01
H62	EVQLVESGGVVVQPGGS<br>LRLSCAASGFTFDDYTM<br>HWVRQAPGKGLEWVSLI<br>SWDGGSTYYADSVKGRF<br>TISRDNSKNSLFLQMNGL<br>RTEDTALYYCAKDILPFN<br>AYYFDYWGQGTLVTVSS	VH3-43	JH4	DIQMTQSPSSLSASVGDR<br>VTITCQGSQDITNYLNW<br>YQQKPGKAPKLLIYDAS<br>NLETGVPSRFSGSGSGTD<br>FTFTISSLQPEDIATYYCQ<br>QYDNLPRLTFGGGTKVEI<br>K	VK1-33	JK4	107.19	103.98	46.79	229	8	96.51
M18	EVQLLESGGGLVQPGGS<br>LRLSCAASGFTFSRYAMS<br>WVRQAPGKGLEWVSAIS<br>GSGGSTYYADSVKGRFTI<br>SRDNSKNTLYLQMNSLR<br>AEDTAVYYCAKGVWFG<br>EEAWGNNWFDPWGQGT<br>LVTVSS	VH3-23	JH5	EIVLTQSPATLSLSPGERA<br>TLSCRASQSVSSYLAWY<br>QQKPGQAPRLLVYDAST<br>RATGVPARFSGSGSGTDF<br>TLTISSLEPEDFAVYYCQ<br>QRSNWPPFTFGPGTKVDI<br>K	VK3-11	JK3	115.74	94.37	42.47	208	11	94.71
M05	QVQLVESGGGVVQPGRS<br>LRLSCAASGFTFSSYAMH<br>WVRQAPGKGLEWVAVIS<br>YDGSNKYYADSVKGRFT<br>ISRDNSKNTLYLQMNSLR<br>AEDTAVYYCARGETYYY<br>DSSGYYEGGYFDYWGQ<br>GTLVTVSS	VH3-30	JH4	EIVMTQSPATLSVSPGER<br>ATLSCRASQSVSSNLAW<br>YQQKPGQAPRWGIYGAS<br>FRATGIPARFSGGGSGTD<br>FILTISSPQAEDSAIYYCQ<br>QFNSWSPLTFGPGTKVHI<br>K	VK3-NL1	JK3	122.62	107.18	48.23	236	8	96.61
L10	QVQLVESGGGVVQPGRS<br>LRLSCAASGFTFSSYGMH<br>WVRQAPGKGLEWVAVIS<br>YDGSNKYYADSVKGRFT<br>ISRDNSKNTLYLQMNSLR<br>AEDTAVYYCAKDLYPVE<br>LATDYNWFDPWGQGTL<br>VTVSS	VH3-30	JH5	EIVLTQSPATLSLSPGERA<br>TLSCRASQSVSSYLAWY<br>QQKPGQAPRLLIYDASN<br>RATGIPARFSGSGSGTDF<br>TLTISSLEPEDFAVYYCQ<br>QRSNWPLTFGGGTKVEI<br>K	VK3-11	JK4	97.45	93.91	42.26	207	8	96.14

Supplementary Table 3 **Amino acid sequences of 42 antibodies and holomogy modeling assessment** (continued)

ID	Sequence information						Verify protein (profiles 3D)			Ramachandran plot		
	VH	IGHV gene	IGHJ gene	VL	IGLV gene	IGLJ gene	Veried score	Ex high	Ex low	Total amino acids	Unbelievable amino acids	Credibility (%)
H02	QVQLVQSGAEVKKPGASVKVSCKASGYTFTSYAMHWVRQAPGQRLEWMGWSNAGNGNTKYSQEFQGRVTITRDTSASTAYMELSSLRSEDTAVYYCARGYSSSWYRGWFDPWGQGTLVTVSS	VH1-3	JH5	DIVMTQTPLSLSVTPGQPASISCKSSQSLLHSDGKTYLYWYLQKPGQWAQLLIYEVSNRFSGVPDRFSGSGSGTDFTLKISRVEAEDVGVYYCMQSIQLPLTFGGGTKVEIK	VK2D-29	JK4	121.86	106.27	47.82	234	6	97.44
M30	EVQLVESGGGLVKPGGSLRLSCAASGFTFSSYSMNWVRQAPGKGLEWVSSISSSSSYIYYADSVKGRFTISRDNAKNSLFLQMSRLRAEDTALYYCAGGEFLGYWGQGTLVTVSS	VH3-21	JH4	QAVVTQDPSLTVSPGGTVTLTCVSSSGAVTSGHYPYWFQQKPGQAPRTLIYDTSNKHSWTPARFSGSLLGGKAALTLSGAQPEDEAEYYCSLLYRGDCVFGGGTKLTVL	VL7-46	JL2	111.5	101.69	45.76	224	8	96.43
M20	EVQLVESGGGLVKPGGSLRLSCAASGFTFSSYSMNWVRQAPGKGLEWVSSISSSSSYIYYADSVKGRFTISRDNAKNSLYLHMSSLRAEDTAIYYCASSGKSSRWYSLYYFDYWGQGTLVTVSS	VH3-21	JH4	QSALTQPASVSGSPGQSITVSCTGTSSDVGSYNLVSWYQQHPGKAPKLMIYEGREAVVSTQSESSSAKSVDTASLTISGLQAEDEADYYCCSYAGRTTLPYVFGTGTKVTVL	VL2-23	JL1	118.7	107.18	48.23	236	9	96.19
M11	QVQLVESGGGLVKPGGSLRLSCAASGFTFSDYYMSWIRQAPGKGLEWVSYISSSGSTIYYADSVIGRFTISRDNAKNSLYLQMNSLRAEDTAVYYCARGLNWNYDYYYYMDVWGKGTTVTVSS	VH3-11	JH6	QSALTQPASVSGSPGQSITISCTGSSSDVGGYNYVSWYQQHPGKAPKLMIYEVSNRPSGVSNRFSGSKSGNTASLTISGLQAEDEADYYCSSYTSSSTLVFGGGTKVTVL	VL2-14	JL2	127.6	105.81	47.61	233	6	97.42
M08	QVQLQQWGAGLLKPSETLSLTCAVYGGSFSGYYWSWIRQPPGKGLEWIGEINHSGSTNYNPSLKSRVTISVDTSKNQFSLKLSSVTAADTAVYYCARGSSIAAAGRRLDYWGQGTLVTVSS	VH4-34	JH4	QSALTQPASVSGSPGQSITISCTGTSSDVGSYNLVSWYQQHPGKAPKLMIYEGSKRPSGVSNRFSGSKSGNTASLTISGLQAEDEADYYCCSYAGRRVFGGGTKLTVL	VL2-23	JL2	129.61	103.98	46.79	229	9	96.07

Supplementary Table 3 **Amino acid sequences of 42 antibodies and holomogy modeling assessment** (continued)

ID	VH	IGHV gene	IGHJ gene	VL	IGLV gene	IGLJ gene	Verify protein (profiles 3D) Veried score	Ex high	Ex low	Ramachandran plot Total amino acids	Unbelievable amino acids	Credibility (%)
M14	QLQLQESGPGLVKPSETLSLTCTVSGGSISSSSYYWGWIRQPPGKGLEWIGSIYYSGSTYYNPSLKSRVTISVDTSKNQFSLKLSSVTAADTAVYYCARHLGGSSGWEADYWGQGTLVTVSS	VH4-39	JH4	QSALTQPASVSGSPGQSITISCTGTSSDVGSYNLVSWYQQHPGKAPKLMIYEGSKRPSGVSNRFSGSKSGNTASLTISGLQAEDEADYYCCSYAGSLVVFGGGTKLTVL	VL2-23	JL2	111.02	92.54	41.64	204	7	96.57
H38	EVQLVQSGAEVKKPGESLRISCKGSGYSFTSYWISWVRQMPGKGLEWMGRIDPSDSYTNYSPSFQGHVTISADKSISTAYLQWSSLKASDTAMYYCARQGEGFDPWGQGTLVTVSS	VH5-a	JH4	QSALTQPASVSGSPGQSITISCTGTSSDVGGYNYVSWYQQHPGKAPKLMIYEVSNRPSGVSNRFSGSKSGNTASLTISGLQAGDEADYYCSSYTSSSTPSVVFGGGTKLTVL	VL2-14	JL2	125.07	103.52	46.58	228	7	96.93
H32	EVQLVQSGAEVKKPGESLRISCKGSGYSFTSYWISWVRQMPGKGLEWMGRIDPSDSYTNYSPSFQGHVTISADKSISTAYLQWSSLKASDTAMYYCARQGEGFDPWGQGTLVTVSS	VH5-a	JH4	QSALTQPASVSGSPGQSITISCTGTSSDVGSYNLVSWYQHQPGKAPKLMIYEGSKRPSGVSNRFSGSRSGNTASLTISGLQAEDEADYYCCSYAGSTTLPYVFGTGTKVTVL	VL2-23	JL1	120.16	103.52	46.58	228	6	97.37
M25	QLQLQESGPGLVKPSETLSLTCTVSGGSISSSSYYWGWIRQPPGKGLEWIGSIYYSGSTYYNPSLKSRVTISVDTSKNQFSLKLSSVTAADTAVYYCARIKYNNNWYEGFDYWGQGTLVTVSS	VH4-39	JH4	SYELTQPPSVSVSPGQTARITCSGDALPKQYAYWYQQKPGQAPVLVIYKDSERPSGIPERFSGSSSGTTVTLTISGVQAEDEADYYCQSVDSSGAYWMFGGGTKLTVL	VL3-25	JL3	127.69	104.89	47.20	231	7	96.97
L05	QVQLQQWGAGLLKPSETLSLTCAVYGGSFSGYYWSWIRQPPGKGLEWIGEINHSGSTNYNPSLKSRVTISVDTSKNQFSLKLSSVTAADTAVYYCARGSSIAAAGRRLDYWGQGTLVTVSS	VH4-34	JH4	QSVLTQPPSASGTPGQRVTISCSGSSSNIGSNPVNWYQQIPGTAPKLLIYSNNQRPSGVPDRFSGSKSGTSASLAISGLQSEDEADYYCAAWDDSLNGVFGGGTKLTVL	VL1-44	JL2	113.32	104.44	47.00	230	5	97.83

Supplementary Table 3 **Amino acid sequences of 42 antibodies and holomogy modeling assessment** (continued)

	Sequence information						Verify protein (profiles 3D)			Ramachandran plot		
ID	VH	IGHV gene	IGHJ gene	VL	IGLV gene	IGLJ gene	Veried score	Ex high	Ex low	Total amino acids	Unbelievable amino acids	Credibility (%)
H23	QLQLQESGPGLVKPSETLSLTCTVSGGSISSSSYYWGWIRQPPGKGLEWIGSIYYSGSTYYNPSLKSRVTISVDTSKNQFSLKLSSVTAADTAVYYCARHLGGSSGWEADYWGQGTLVTVSS	VH4-39	JH4	QSVLTQPPSVSEAPRQRVTISCSGSSSNIGNNAVNWYQQLPGKAPKLLIYYDDLLPSGVSDRFSGSKSGTSASLAISGLQSEDEADYYCAAWDDSLNGPVFGTGTKVTVL	VL1-36	JL1	118.95	105.35	47.41	232	7	96.98
H55	QLQLQESGPGLVKPSETLSLTCTVSGGSISSSSYYWGWIRQPPGKGLEWIGSIYYSGSTYYNPSLKSRVTISVDTSKNQFSLKLSSVTAADTAVYYCARQISSTSLTSKRYFDLWGRGTLVTVSS	VH4-39	JH2	QSVLTQPPSASGTPGQRVTISCSGSSSNIGSNYVYWYQQLPGTAPKLLIYRNNQRPSGVPDRFSGSKSGTSASLAISGLRSEDEADYYCAAWDDSLSGPVFGGGTKLTVL	VL1-47	JL2	115.34	106.73	48.03	235	8	96.60
H01	QVQLVQSGAEVKKPGASVKVSCKASGYTFTSYDINWVRQATGQGLEWMGWMNPNSGNTGYAQKFQGRVTMTRNTSISTAYMELSSLRSEDTAVYYCARGYSSSWYRGWFDPWGQGTLVTVSS	VH1-8	JH5	SYELTQPPSVSVSPGQTASITCSGDKLGDKYACWYQQKPGQSPVLVIYQDSKRPSGIPERFSGSNSGNTATLTISGTQPMDEADYYCQAWDNTTAVFGGGTRLSVL	VL3-1	JL2	100.35	92.08	41.44	203	1	99.51
H48	QVQLVESGGGVVQPGRSLRLSCAASGFTFSSCGMYWVRQSPGKGLEWVAVISYDGSNKYYADSVKGRFTISRDNSKNTLYLQMNTLRPEDTAVYYCAKDIWMIYSDDSGPIDFWGQGTLVTVSS	VH3-30	JH4	SYELTQPPSVSVSPGQTASITCSGDKLGDKYACWYQQKPGQSPVLVIYQDSKRPSGIPERFSGSNSGNTATLTISGTQAMDEADYYCQAWDSSTSYVFGTGTKVTVL	VL3-1	JL1	112.96	104.89	47.20	231	6	97.40
H58	EVQLVESGGGLVKPGGSLRLSCAASGFTFSNAWMSWVRQAPGKGLEWVGRIKTQSDGATTTYAAPVKGRFIISRDDSKNMVYLELNSLNAEDTAVYYCSTGPNYFYDSGGFKYRDKWGQGTLVTVSS	VH3-15	JH4	SYELTQPPSVSVSPGQTASITCSGDKLGDKYACWYQQKPGQSPVLVIYQDSKRPSGIPERFSGSNSGNTATLTISGTQPMDEADYYCQAWGTTSDQVVFGGGTKLTVL	VL3-1	JL2	121.89	106.73	48.03	235	8	96.60

Supplementary Table 3 **Amino acid sequences of 42 antibodies and holomogy modeling assessment** (continued)

	Sequence information						Verify protein (profiles 3D)			Ramachandran plot		
ID	VH	IGHV gene	IGHJ gene	VL	IGLV gene	IGLJ gene	Veried score	Ex high	Ex low	Total amino acids	Unbelievable amino acids	Credibility (%)
H26	QVQLVESGGGVVQPGRS<br>LRLSCAASGFGFSSYALH<br>WVRQAPGKGLEWVAVIS<br>YDGSNKYYADSVKGRFT<br>ISRDNSKNTLYLQMNSLR<br>PEDTAVYFCARDYCSGG<br>SCHCDHWGQGTQVTVSS	VH3-30	JH4	QSVLTQPPSASGTPGQRV<br>TISCSGSSSNIGRNTVNW<br>YQQLPGTAPKLLIYSNNQ<br>RPSGVPDRFSGSKSGTSA<br>SLAISGLQSEDDTDYYCA<br>AWDDSLNGVVFGGGTK<br>LTVL	VL1-44	JL2	119.45	104.89	47.20	231	6	97.40
M01	QVQLVQSGAEVKKPGAS<br>VKVSCKASGYTFTSYAM<br>HWVRQAPGQGLEWMGII<br>NPSGGSTSYAQKFQGRV<br>TMTRDTSTSTVYMELSSL<br>RSEDTAVYYCARGYSSS<br>WYRGWFDPWGQGTLVT<br>VSS	VH1-46	JH5	QSALTQPASVSGSPGQSI<br>TISCTGTSSDVGAYNFVS<br>WYQQHPGKAPKLMIYEV<br>SNRPSGVSNRFSGSKSAN<br>TASLTISGLQAADEADYY<br>CSSYTSSSTWVFGGGTK<br>VTVL	VL2-14	JL3	128.88	105.35	47.41	232	6	97.41
L23	QVQLVQSGAEVKKPGAS<br>VKVSCKASGYTFTGYYM<br>HWVRQAPGQGLEWMG<br>WINPNSGGTNYAQKFQG<br>RVTMTRDTSISTAYMELS<br>RLRSDDTAVYYCARGIA<br>VASWFDPWGQGTLVTVS<br>S	VH1-2	JH5	QSALTQPASVSGSPGQSI<br>TLGVAGTSSDVGGYTYV<br>SWYQQHPGKAPKLMIYE<br>VSNRPSGVSNRFSGSKSG<br>NTASLTISGLQADDETDY<br>YCSSYSSSSSRVVFGGGT<br>KVTVL	VL2-14	JL2	118.64	104.44	47.00	230	7	96.96
H20	QVQLVQSGAEVKKPGAS<br>VKVSCKASGYTFTSYYM<br>HWVRQAPGQGLEWMGII<br>NPSGGSTSYAQKFQGRV<br>TMTRDTSTSTDYMELSSL<br>RSEDTAVYYCARDLRQT<br>GNDYWGQGTLVTVSS	VH1-46	JH4	QSVLTQPPSVSGAPGQRV<br>TISCTGSSSNIGAGPEAVG<br>INAESGTAPKLGIYGNSN<br>RPSGVPDRFSGSKSGTSA<br>SLAITGLQAEDEADYYC<br>QSYDSSREVFGGGTKLT<br>VL	VL1-40	JL2	111.22	103.06	46.38	227	10	95.59
H52	QVQLVQSGAEVKKPGAS<br>VKVSCKASGYTFTSYGIS<br>WVRQAPGQGLEWMGWI<br>SAYNGNTNYAQKLQGR<br>VTMTTDTSTSTAYMELR<br>SLRSDDTAVYYCAREPP<br>WYFDSSGSYGRDNWFDP<br>WGQGTLVTVSS	VH1-18	JH5	QSVLTQPPSVSGAPGQRV<br>TISCTGSSSNIGAGYDVH<br>WYQHLPGTAPKLLIYGN<br>SNRPSGVPDRFSGSKSGT<br>SASLAITGLQAEDEAEYY<br>CQSYDSSLSGSRDVFGTG<br>TKVTVL	VL1-40	JL1	120.44	109.93	49.47	242	7	97.11

Supplementary Table 3 **Amino acid sequences of 42 antibodies and holomogy modeling assessment** (continued)

	Sequence information						Verify protein (profiles 3D)			Ramachandran plot		
ID	VH	IGHV gene	IGHJ gene	VL	IGLV gene	IGLJ gene	Veried score	Ex high	Ex low	Total amino acids	Unbelievable amino acids	Credibility (%)
H07	QVQLVQSGAEVKKPGAS<br>VKVSCKASGYTFTSYDIN<br>WVRQATGQGLEWMGW<br>MNPNSGNTGYAQKFQG<br>RVTMTRNTSISTAYMELS<br>SLRSEDTAVYYCARGYS<br>SSWYRGWFDPWGQGTL<br>VTVSS	VH1-8	JH5	QSVLTQPPSASGTPGQRV<br>TISCSGSSSNIGSNTVNW<br>YQQLPGTAPKLLIYSNNQ<br>RPSGVPDRFSGSKSGTSA<br>SLAISGLQSEDEADYYCA<br>AWDDSLNGYVFGTGTK<br>VTVL	VL1-44	JL1	126.32	105.35	47.41	232	5	97.84
H45	QVQLVQSGAEVKKPGAS<br>VKVSCKASGYTFTSYGIS<br>WVRQAPGQGLEWMGWI<br>SAYNGNTNYAQKLQGR<br>VTMTTDTSTSTAYMELR<br>SLRSDDTAVYYCARVVD<br>IVVVPAHMDVWGQGTT<br>VTVSS	VH1-18	JH3	QSVLTQPPSVSAAPGQK<br>VTISCSGSSSNIGNNYVS<br>WYQQLPGTAPKLLIYDN<br>NKRPSGIPDRFSGSKSGT<br>SATLGITGLQTGDEADY<br>YCGTWDSSLSAGVFGGG<br>TKLTVL	VL1-51	JL2	110.3	105.35	47.41	232	9	96.12

VH: variable region of heavy chain; VL: variable region of light chain; Ex: expected.

Supplementary Table 4 **Properties of the expressed antibodies**

		Fv	HCDR3					LCDR3				
Cluster	ID	Energy (kcal/mol)	Energy (kcal/mol)	Length	Alkaline	Polar	Acidic	Energy (kcal/mol)	Length	Alkaline	Polar	Acidic
1	H40*	–108.16	–32.45	16	2	6	2	–6.71	10	0	8	0
	L18	–105.99	0.00	14	2	5	1	0.00	8	1	5	0
	Average	–91.16	–11.80	15.75	2.50	6.63	1.38	–3.72	9.00	0.63	6.38	0.00
2	M08	–61.58	–0.01	15	3	5	1	0.00	8	2	4	0
	M25*	–86.95	–4.60	15	2	7	2	0.00	11	0	6	1
	Average	–69.89	–7.72	15.00	2.50	6.17	1.50	–2.45	10.00	0.33	4.00	1.17
3	H45*	–82.72	–41.69	15	2	0	2	0.00	11	0	6	1
	H52*	–132.98	–53.09	22	2	8	4	–0.01	13	1	8	2
	H20	–93.10	–11.92	11	2	5	2	–0.03	9	1	5	2
	Average	–77.29	–18.43	13.80	1.70	4.90	1.70	–4.50	10.70	0.30	6.40	0.80
4	M20*	–108.21	–56.49	17	2	10	1	–0.05	12	1	7	0
	M30	–9.53	–1.89	8	0	4	1	18.18	9	1	4	1
	Average	–80.89	–13.47	15.00	1.67	7.33	2.00	1.09	10.50	0.33	5.67	0.83
5	M27	–108.63	–0.01	14	2	3	4	0.00	10	0	6	0
	M05*	–99.36	–29.33	21	1	14	4	–1.04	10	0	6	0
	Average	–97.61	–17.99	17.40	1.20	5.80	3.60	–2.02	9.40	0.40	6.00	0.00
6	H47*	–84.54	–30.56	19	2	8	3	–17.09	10	0	7	0
	M13	–92.32	–30.05	16	1	8	2	–9.37	9	0	7	0
	Average	–100.91	–12.21	16.00	1.00	8.00	2.00	–6.86	9.57	0.43	6.57	0.14
All	Average	–85.74	–13.84	15.43	1.86	6.19	1.95	–3.36	9.90	0.40	5.93	0.50

Antibodies marked with star are elite ones, while others are control. Fv: fragment of variable region; HCDR3: heavy chain complementarity determining region 3; LCDR3: light chain complementarity determining region 3.
